# #Covid4Rheum: an analytical twitter study in the time of the COVID-19 pandemic

**DOI:** 10.1007/s00296-020-04710-5

**Published:** 2020-09-29

**Authors:** Nikolas Ruffer, Johannes Knitza, Martin Krusche

**Affiliations:** 1 Department of Rheumatology and Immunology, Klinikum Bad Bramstedt, Bad Bramstedt, Germany; 2 Department of Internal Medicine 3-Rheumatology and Immunology, Friedrich-Alexander-Universität Erlangen-Nürnberg, Universitätsklinikum Erlangen, Erlangen, Germany; 3 Department of Rheumatology and Clinical Immunology, Charité-Universitätsmedizin Berlin, Charitéplatz 1, 10117 Berlin, Germany

**Keywords:** Twitter, SARS-CoV-2, COVID-19, Rheumatology, Hashtag

## Abstract

Social media services, such as Twitter, offer great potential for a better understanding of rheumatic and musculoskeletal disorders (RMDs) and improved care in the field of rheumatology. This study examined the content and stakeholders associated with the Twitter hashtag #Covid4Rheum during the COVID-19 pandemic. The content analysis shows that Twitter connects stakeholders of the rheumatology community on a global level, reaching millions of users. Specifically, the use of hashtags on Twitter assists digital crowdsourcing projects and scientific collaboration, as exemplified by the COVID-19 Global Rheumatology Alliance registry. Moreover, Twitter facilitates the distribution of scientific content, such as guidelines or publications. Finally, digital data mining enables the identification of hot topics within the field of rheumatology.

**Electronic supplementary material:**

The online version of this article (10.1007/s00296-020-04710-5) contains supplementary material, which is available to authorized users.

## Introduction

The use and influence of social media in rheumatology have increased rapidly in recent years [1]. Via the use of hashtags (#), specific topics, individual persons or institutions, as well as projects and ideas, can be identified and linked to each other and therefore foster rapid digital exchange.

Social media is frequently used in the scientific rheumatology community to exchange and discuss information and opinions [2], and to promote meetings [3], projects or publications [3, 4]. Social media channels can, furthermore, be useful for expanding education and research perspectives [5]. However, social media can be abused to broadcast misinformation, unethical promotion of content, and can enable unprofessional behavior. Therefore, the role of social media editing is becoming increasingly important. This includes not only filtering and promoting credible and expert-proven information, but also the activity of ethical guidance [6].

In the context of the COVID-19 pandemic, Twitter played a prominent role in moderating the scientific discourse, with subsequent implementation of research projects [7]. New COVID-19-associated hashtags were created and frequently used by various stakeholders in the rheumatological community. For example, the hashtag #Covid4Rheum was first introduced by @ACR_Journals on March 13th 2020 and became an influential hashtags connecting COVID-19 and rheumatology related topics, such as the use of immunosuppressive drugs, the implementation of telemedicine, as well as the establishment and data distribution of the COVID-19 Global Rheumatology Alliance [8].

Applying digital data mining and crowdsourcing to social media offers new opportunities for scientific research [9, 10]. Via the use of data and text mining—a technology which can be applied to extract potentially valuable knowledge from large data sets [11]- all Tweets related to #Covid4Rheum were identified and analysed.

## Objective

To examine the influence, content and stakeholders associated with the Twitter hashtag #Covid4Rheum during the COVID-19 pandemic.

## Methods

A two-step hashtag analysis of tweets associated with #Covid4Rheum between 13/03/2020 and 01/06/2020 was carried out. First, the Twitter analytics tool *Vicinitas* (https://vicinitas.io) provided basic tweet data (i.e. language), and calculated the engagement (the sum of intentional interactions in response to a post with a tracked hashtag) and influence (sum of followers of the tweet creators) of the tweets.

In a second step, two independent reviewers analysed the primary tweets (re-tweets and replies were excluded) for qualitative content analysis. Disagreement was resolved by consultation of a third independent reviewer.

Twitter user and content analysis definitions are illustrated in the Supplementary material A.

## Results

The study period covers 2483 tweets (245 primary tweets, 2201 retweets, 37 replies) from 1397 users in total. Overall, #Covid4Rheum reached an engagement in 6916 users and an influence of 6,725,773 users. A total of 242 (9.8%) primary tweets from 78 users (19 different countries, 7 different languages) were included for content analysis. Most tweets were created by healthcare professionals and/or researchers (110/245; 44.9%), whereas patients (5/245; 2%) or support groups (16/245; 6.5%) rarely engaged with the hashtag. The vast majority of the primary tweets were created by professional Twitter users (218/245; 88.97%). English was the predominant language (206/228; 90.3%). Basic tweet characteristics are displayed in Table 1.

**Table 1:** Basic tweet characteristics

Tweet creators	Tweets	Percent
Healthcare professional/researcher	110	44.9 (110/245)
Scientific society	80	32.7 (80/245)
Scientific journal	21	8.6 (21/245)
Support group	16	6.5 (16/245)
Research group	7	2.9 (7/245)
Other	6	2.4 (6/245)
Patient	5	2.0 (5/245)

Geolocation	Tweets^a^	Percent^a^
Argentina	1	0.4 (1/225)
Australia	2	0.9 (2/225)
Canada	6	2.7 (6/225)
Columbia	4	1.8 (4/225)
Germany	9	4.0 (9/225)
India	4	1.8 (4/225)
Ireland	1	0.4 (1/225)
Jordan	7	3.1 (7/225)
Norway	4	1.8 (4/225)
Pakistan	2	0.9 (2/225)
Peru	4	1.8 (4/225)
Philippines	2	0.9 (2/225)
Qatar	1	0.4 (1/225)
Saudi Arabia	3	1.3 (3/225)
Spain	3	1.3 (3/225)
Turkey	1	0.4 (1/225)
United Arab Emirates	3	1.3 (3/225)
United Kingdom	18	8.0 (18/225)
United States	150	66.7 (150/225)

Language^b^	Tweets^c^	Percent^c^
English	206	90.3 (206/228)
German	9	3.9 (9/228)
Spanish	8	3.5 (8/228)
Arabian	4	1.7 (4/228)
Norwegian	4	1.7 (4/228)
Japanese	1	< 1 (1/228)
Turkish	1	< 1 (1/228)

^a^Geolocation was not available for all tweet creators

^b^Language was not available for all tweets (e.g. tweet exclusively comprised hashtags)

^c^Tweets could include multiple languages

A significant proportion of the tweets (102/242; 42.1%) referred to the COVID-19 Global Rheumatology Alliance registries and encouraged the participation of both healthcare professionals and patients (72/102; 70.5%). Specific rheumatic and musculoskeletal diseases (RMD) were mentioned in a minor proportion of tweets (18/242; 7.4%) (Table 2). Approximately a quarter (64/242; 26.4%) of the tweets discussed specific therapeutic agents for the management of RMDs or COVID-19. Hydroxychloroquine/chloroquine (41 tweets) and tocilizumab (7 tweets) were the most frequently mentioned drugs (Table 2).

**Table 2:** Tweet content analysis

Rheumatic and musculoskeletal diseases (RMD)	Tweets^a^	Percent^a^
Spondyloarthritis	8	44.4 (8/18)
Systemic lupus erythematodes	8	44.4 (8/18)
Rheumatoid Arthritis	7	38.3 (7/18)
Psoriatic arthritis	6	33.3 (6/18)
Sjögren’s syndrome	2	11.1 (2/18)
Vasculitis	2	11.1 (2/18)
Pediatric conditions	2	11.1 (2/18)
Antiphospholipid syndrome	1	5.5 (1/18)
Systemic sclerosis	1	5.5 (1/18)

Therapeutic agents^b^	Tweets^b^	Percent^b^
Hydroxychloroquine/chloroquine	41	64.1 (41/64)
Tocilizumab	7	10.9 (7/64)
Methotrexate	3	4.6 (3/64)
Sarilumab	3	4.6 (3/64)
Steroids	3	4.6 (3/64)

Management of RMD patients without COVID-19	Tweets	Percent
Guideline/recommendation (scientific society)	23	53.4 (23/43)
Recommendation (healthcare professional)	17	39.5 (17/43)
Other	3	6.9 (3/43)

Management of RMD patients with COVID-19	Tweets	Percent
Guideline/recommendation (scientific society)	9	60.0 (9/15)
Recommendation (healthcare professional)	5	33.3 (4/15)
Other	1	6.6 (1/15)

^a^Tweets could include multiple conditions

^b^Tweets could include multiple therapeutic agents

Interestingly, tweets infrequently referred to the special challenges of the COVID-19 pandemic for the management of RMD patients without (43/242; 17.7%) or with COVID-19 infection (15/242; 6.1%) (Table 2): However, the use of telehealth (27/242; 11.1%) was a common theme for the management of RMD patients in the current situation. Notably, only 9 (9/242; 3.7%) tweets addressed patient education in the context of the pandemic. Finally, a third (81/242; 33.4%) of the tweets contained supplementary material, such as resources published by scientific societies (24/242; 9.9%) or scientific journals (32/242; 13.2%).

## Discussion

By using Twitter and connecting topics to a specific hashtag it is possible to effectively reach a global audience. Our analysis showed that a relatively small number of the 245 primary tweets which used the hashtag #Covid4Rheum were able to reach an overall engagement in 6916 users and influence of over 6.7 million. Although English was the predominant language used (90.3%), tweets were in 7 different languages and tweet creators were located in 19 different countries, demonstrating that Twitter can reach and connect the global rheumatology community (Table 1). It is noteworthy that the majority of tweets originated from anglophone countries (177/225; 78.6%). This may be due to the overrepresentation of Twitter users in these countries, or resultant of the language barrier for users from other countries.

**Fig. 1:**
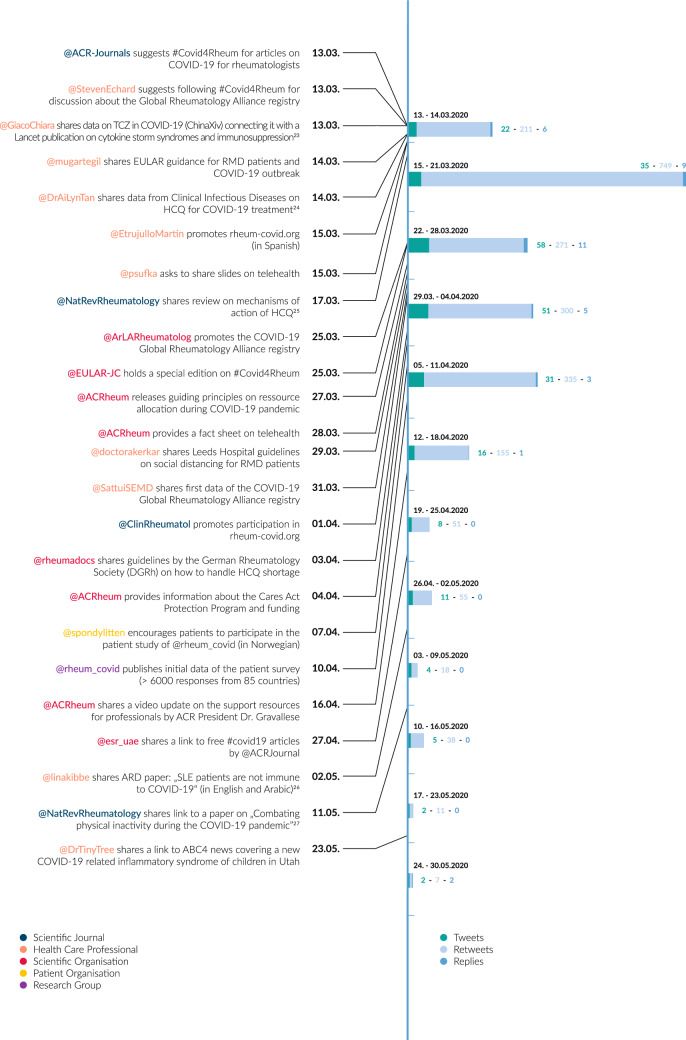
Tweet timeline and tweet content examples [23–27]

Notably, various groups, such as healthcare professionals (HCP), support groups (i.e. LUPUS UK), scientific organizations (i.e. ACR) and scientific journals (i.e. *Nature Reviews Rheumatology*), engaged with the hashtag. Interestingly, the content analysis showed that different stakeholders of the rheumatology community used Twitter in connection with #Covid4Rheum for various reasons. For example, HCP shared knowledge and experiences concerning COVID-19 related topics, scientific societies were promoting guidelines or health policy-related content, and scientific journals shared and promoted publications (Fig. 1).

Importantly, only few patients (2.9%) and support groups (6.5%) engaged with the hashtag, although several tweets encouraged patient participation in the COVID-19 Global Rheumatology Alliance registry. The authors presume that Twitter is less attractive for patients, possibly due to the short message system, in comparison to other social media platforms, such as Facebook or Instagram, which may be more likely to be used by this cohort.

At the beginning of the COVID-19 pandemic, there was a significant degree of uncertainty regarding the treatment of RMD patients. By applying digital crowdsourcing, the COVID-19 Global Rheumatology Alliance was rapidly set up [12]. Via Twitter and the use of the specific hashtags such as #Covid4Rheum, collaborations and enrolment of patients were actively promoted. 42.1% of the tweets which used this hashtag referred to the COVID-19 Global Rheumatology Alliance registry and a majority of these tweets encouraged participation (72/102; 70.5%). #Covid4Rheum was used by HCPs, journals and scientific organizations to promote the registry and to share preliminary results and updates. The data from the COVID-19 Global Rheumatology Alliance significantly contributed to the scientific discourse on RMDs and COVID-19. As a result, first major publications were published [13, 14].

The analysis found that 26.4% of the tweets were related to immunosuppressive agents in the context of the COVID-19 pandemic. The main issues were the presumed vulnerability of RMD patients for COVID-19 infection due to immunosuppressive therapy, the management of the hydroxychloroquine shortage and the potential use of immunosuppressive treatment for COVID-19 infections. The results illustrate the importance of these topics for the rheumatological community during the COVID-19 pandemic [15]. Furthermore, they underline the potential of Twitter to foster the exchange of medical knowledge (“from Twitter to bedside”).

The COVID-19 pandemic and the wide range of quarantine and security precautions have greatly affected the healthcare for many RMD patients. In particular, telemedicine was rapidly introduced by many clinics to deal with this development [16, 17]. Interestingly, this is also reflected in our analysis of the tweets. In total, 11.1% of the tweets were related to the topic of telemedicine, in particular the requirements for the setup, the implementation and reimbursement.

Naturally, social media is very dynamic, as discussions and interpretation of facts can sometimes change rapidly. In particular, unreliable data sets and flawed publications can cause great reputational damage, and indirectly harm patients via uncritical dissemination of false information, as the recent retractions of COVID-19 related publications in *The Lancet* [18] and *The New England Journal of Medicine* [19] have illustrated.

The limited period of this hashtag analysis and the focus on primary tweets are limitations of this study. Particularly by tweet commenting, the content can be interpreted differently, or debates can be redirected. Likewise, new scientific findings and medical developments can shift the focus of the tweets in another direction, so that an over-representation of certain topics cannot be ruled out. Therefore, analyses of social media content should always be subject to a thorough review and be interpreted within the time frame of its creation.

This analysis highlights the wide range of opportunities for the use of social media in rheumatology: Digital platforms like Twitter are able to engage with a vast audience and connect different members of the rheumatology community. As Nikipharou et al. [20] already pointed out, social media platforms provide novel ways for opinion sharing, learning and development, which is especially important in times of uncertainties during a pandemic. Notably, social media can effectively increase the impact of scientific publications which might be of interest to researchers and scientific journals alike [21]. Social media can direct attention and increase participation for digital crowdsourcing projects, such as the COVID-19 Global Rheumatology Alliance registries or healthcare policy projects.

Our analysis underlines the feasibility of digital data mining for the identification and analysis of hashtag related content on Twitter. Data mining techniques might also be applied to other social media platforms and rheumatology related topics to gain more insight into the content and related stakeholders. As Pérez-Pérez et al. [22] suggested, Twitter content analysis might even support decision making among health-related stakeholders.

The potential of social media should progressively be unlocked to enhance communication between all members of the rheumatology community, in particularly with RMD patients, and to enable the setup and participation in research and healthcare-related topics to improve treatment and management of RMDs.

In conclusion, social media, Twitter in particular, effectively connects members of the rheumatology community on a global scale, allowing for rapid content-sharing, communication and scientific cooperation (digital crowdsourcing), as this study shows. Moreover, digital data mining of social media content can be used to identify hot topics and blind spots within the field of rheumatology.

## Pre-publication archiving

This manuscript has not been subject to pre-publication archiving.

## Electronic supplementary material

Below is the link to the electronic supplementary material.

Supplementary file1 (DOCX 22 kb)

Supplementary file2 (XLSX 5936 kb)

Supplementary file3 (XLSX 196 kb)
