# Supplementary material for: #Covid4Rheum: an analytical twitter study in the time of the COVID-19 pandemic
Source: Rheumatol Int. 2020 Sep 29;40(12):2031–7. doi: 10.1007/s00296-020-04710-5 (PMC7523492; doi:10.1007/s00296-020-04710-5)
Supplement: Supplementary file 1 — Supplementary file1 (DOCX 22 kb) [file 296_2020_4710_MOESM1_ESM.docx]

## Twitter users

**Tweet creator**: Who created the tweet?

Tweet creators (screen name) were categorized: (1) scientific journal, (2) scientific society, (3) healthcare professional / researcher, (4) patient, (5) relative of patient, (6) support group, (7) research group (university or clinic), (8) pharmaceutical company and (9) other business. Tweet creators that did not fit into the mentioned categories were characterized as (10) ‘other’.

**Geolocation**: Where is the tweet creator located?

Geolocation of tweet creators were noted if provided in the self-description. In addition, webpages of the tweet creators were accessed if reported.

## Content analysis

**COVID-19 Global Rheumatology Alliance registries**: Does the tweet mention the *COVID-19 Global Rheumatology Alliance registries* ([www.rheum-covid.org](http://www.rheum-covid.org))?

If the tweet did not refer to the registry, it was characterized as (1) ‘no’. Tweets that mentioned the registry (i.e. tweet included the Twitter username of the registry [@rheum_covid], link to webpage of the registry or figure with clinical data from the registry) were characterized as (2) ‘yes’.

*– encouragement to participate in the Global Rheumatology Alliance physician-reported registry*: The tweet encourages healthcare professionals to provide clinical data of RMD patients for the registry.

*– data from the Global Rheumatology Alliance physician-reported registry*: The tweet reports data from the registry.

**Specific RMD**: Does the tweet mention specific rheumatic and musculoskeletal diseases (RMD)?

If the tweet did not refer to specific RMDs, it was characterized as (1) ‘no’. Tweets that mentioned specific RMDs or their commonly used abbreviations (i.e. ‘RA’ for rheumatoid arthritis, ‘SLE’ or ‘lupus’ for systemic lupus erythematodes or ‘SSc’ for systemic sclerosis) were characterized as (2) ‘yes’. In addition, the specific RMDs were noted for further analysis if applicable.

**Therapeutic agents**: Does the tweet mention a specific therapeutic agent that is commonly used for the treatment of RMDs (i.e. hydroxychloroquine, rituximab, tocilizumab)?

If the tweet did not refer to a specific therapeutic agent, it was characterized as (1) ‘no’. Tweets that mentioned specific therapeutic agents or their commonly used abbreviations (i.e. ‘HCQ’ for hydroxychloroquine) were characterized as (2) ‘yes’. In addition, the specific therapeutic agents were noted for further analysis if applicable.

**Management of RMD patients without active or past COVID-19 infection**: Does the tweet refer to the management of RMD patients *without* active or past COVID-19 infection in the context of the special circumstances of the COVID-19 pandemic?

If the tweet did not refer to the management of RMD patients *without* active or past COVID-19 infection, it was characterized as (1) ‘no’. Tweets that addressed specific problems (i.e. access to healthcare providers, guidance to ongoing treatment in RMD patients, precautions for RMD patients due to immunosuppressive therapy) regarding the management of RMD patients *without* active or past COVID-19 infection were characterized as (2) ‘yes’ (i.e. guidelines or recommendations from scientific societies, recommendations form individual healthcare professionals, use of telehealth during the COVID-19 pandemic). In addition, the content of the tweet was characterized by subcategories:

*– guidelines or recommendations for the management of RMD patients (scientific society)*: The tweet (mainly refers to guidelines or recommendations from a scientific society (i.e. American College of Rheumatology). Guidelines or recommendations include statements that address a specific clinical problem.

– *recommendations for the management of RMD patients (individual healthcare professionals, researchers, research groups)*: The tweet reflects personal recommendations or viewpoints for the management of RMD patients during the COVID-19 pandemic from individual healthcare professionals, researchers or research groups.

– other: Tweets that did not fit into the mentioned subcategories were characterized as ‘other’.

**Management of RMD patients with active COVID-19 infection**: Does the tweet refer to the management of RMD patients *with* active COVID-19 infection?

If the tweet did not refer to the management of RMD patients *with* active COVID-19 infection, it was characterized as (1) ‘no’. Tweets that addressed the management of RMD patients *with* active COVID-19 infection were characterized as (2) ‘yes’ (i.e. guidelines or recommendations from scientific societies, advice/viewpoint from healthcare professionals or researchers). In addition, the content of the tweet was characterized by subcategories:

*– guidelines or recommendations for the management of RMD patients with active COVID-19 infection (scientific society)*: The tweet refers to guidelines or recommendations from a scientific society (i.e. American College of Rheumatology). Guidelines or recommendations include statements that address a specific clinical problem.

– *recommendations for the management of RMD patients with active COVID-19 infection (individual healthcare professionals, researchers, research groups)*: The tweet reflects personal recommendations or viewpoints for the management of RMD patients with active COVID-19 infection from individual healthcare professionals, researchers or research groups (i.e. advice/viewpoint regarding therapeutic strategies, application of specific therapeutic agents).

– other: Tweets that did not fit into the mentioned subcategories were characterized as ‘other’.

**Patient education**: Does the tweet refer to the education of patients without active COVID-19 infection regarding the SARS-CoV-2 virus, COVID-19 infection or health-related behaviour in the context of the COVID-19 pandemic?

If the tweet did not refer to the education of RMD patients, it was characterized as (1) ‘no’. Tweets that aimed to support the education of RMD patients were characterized as (2) ‘yes’ (i.e. educating RMD patients about personal precautions in the context of the COVID-19 pandemic).

**Clinical data from patients with COVID-19 infection**: Does the tweet refer to clinical data from patients with active COVID-19 infection?

If the tweet did not refer to clinical data from patients with active COVID-19 infection, it was characterized as (1) ‘no’. Tweets that specifically report clinical data (i.e. clinical descriptions of disease manifestations, transmission of COVID-19 infection or management of patients with active COVID-19 infection) from patients with active COVID-19 infection were characterized as (2) ‘yes’. In addition, the content of the tweet was characterized by subcategories:

*– RMD patients with active COVID-19 infection*: The tweet referred to clinical data from primary studies such as case series, clinical trials or registries (such as the *Global Rheumatology Alliance physician-reported registry*).

– *Patients without RMDs and active COVID-19 infection*: The tweets referred to clinical data from primary studies such as case series, clinical trials or registries.

**SARS-CoV-2 virus**: Does the tweet refer to basic research regarding the SARS-CoV-2 virus?

If the tweet did not refer to basic research regarding the biology of the SARS-CoV-2 virus, it was characterized as (1) ‘no’. Tweets that specifically address basic aspects of SARS-CoV-2 virus’ biology (i.e. replication process, in vitro reaction to therapeutic agents) were characterized as (2) ‘yes’.

**Patient survey**: Does the tweet refer to a patient survey?

If the tweet did not refer to a patient survey, it was characterized as (1) ‘no’. Tweets that referred to data from surveys in RMD patients or encouraged RMD patients to report their personal experience with the COVID-19 pandemic were characterized as (2) ‘yes’. In addition, the content of the tweet was characterized by subcategories:

*– RMD patients with active or past COVID-19 infection*: The tweet referred to a patient survey in RMD patients with active or past COVID-19 infection.

– *Patients without RMDs and active or past COVID-19 infection*: The tweet referred to a patient survey in patients without RMDs and active or past COVID-19 infection.

**Attached resources**: Does the tweet refer to a specific publication?

If the tweet did not refer to a specific publication, it was characterized as (1) ‘no’. Tweets that referred to a specific publication were characterized as (2) ‘yes – peer reviewed’ or (3) ‘yes – open preprint’ or (4) ‘other’ if the previous categories were not applicable. In addition, the content of the tweet was characterized by subcategories:

*– case report or case series*

*– comment*

*– guidelines / recommendations from a scientific society*

*– clinical study*

*– other resources from a scientific society*

*– review*

*– basic research*

**Telehealth**: Does the tweet refer to use of telehealth in the context of the COVID-19 pandemic?

If the tweet did not refer to the use of telehealth in the context of the COVID-19 pandemic, it was characterized as (1) ‘no’. Tweets that shared information about the use of telehealth, were characterized as (2) ‘yes’ (i.e. personal experiences with the use of telehealth, publications that discuss the role of telehealth during the COVID-19 pandemic).

**Mobile application**: Does the tweet refer to use of mobile applications in the context of the COVID-19 pandemic?

If the tweet did not refer to the use of mobile applications in the context of the COVID-19 pandemic, it was characterized as (1) ‘no’. Tweets that shared information about the use of mobile applications, were characterized as (2) ‘yes’ (i.e. specific applications with focus on medical usage mentioned).

**Promoting events**: Does the tweet promote any specific event?

If the tweet did not promote any specific event, it was characterized as (1) ‘no’. Tweets that promoted specific events such as webinars or conferences were characterized as (2) ‘yes’. In addition, the specific event was noted, if reported.
